# Spatiotemporal AMPKα2 deletion in mice induces cardiac dysfunction, fibrosis and cardiolipin remodeling associated with mitochondrial dysfunction in males only

**DOI:** 10.1186/s13293-021-00394-z

**Published:** 2021-09-17

**Authors:** Lucile Grimbert, Maria-Nieves Sanz, Mélanie Gressette, Catherine Rucker-Martin, Marta Novotova, Audrey Solgadi, Ahmed Karoui, Susana Gomez, Kaveen Bedouet, Eric Jacquet, Christophe Lemaire, Vladimir Veksler, Mathias Mericskay, Renée Ventura-Clapier, Jérôme Piquereau, Anne Garnier

**Affiliations:** 1 Faculté de Pharmacie, UMR-S1180, INSERM, Université Paris-Saclay, 5 rue J-B Clément, 92296 Châtenay-Malabry, France; 2 Université Paris-Saclay, Inserm, Hypertension Artérielle Pulmonaire: Physiopathologie et Innovation Thérapeutique, 92350 Le Plessis Robinson, France; 3 Department of Cellular Cardiology, Institute of Experimental Endocrinology, Biomedical Research Center, University Science Park for Biomedicine, Slovak Academy of Sciences, Bratislava, Slovakia; 4 Service d’Analyse des Médicaments et Métabolites, Université Paris-Saclay, Inserm, CNRS, Institut Paris Saclay d’Innovation Thérapeutique, 92296 Châtenay-Malabry, France; 5 Université Paris-Saclay, CNRS, Institut de Chimie Des Substances Naturelles, UPR 2301, 91198 Gif-sur-Yvette, France; 6 Université Versailles St-Quentin, Université Paris-Saclay, Inserm, UMR-S 1180, 92296 Châtenay-Malabry, France

**Keywords:** Heart, AMP-activated protein kinase, Fibrosis, Cardiolipins, Energy metabolism

## Abstract

**Background:**

The AMP-activated protein kinase (AMPK) is a major regulator of cellular energetics which plays key role in acute metabolic response and in long-term adaptation to stress. Recent works have also suggested non-metabolic effects.

**Methods:**

To decipher AMPK roles in the heart, we generated a cardio-specific inducible model of gene deletion of the main cardiac catalytic subunit of AMPK (*Ampkα2*) in mice. This allowed us to avoid the eventual impact of AMPK-KO in peripheral organs.

**Results:**

Cardio-specific *Ampkα2* deficiency led to a progressive left ventricular systolic dysfunction and the development of cardiac fibrosis in males. We observed a reduction in complex I-driven respiration without change in mitochondrial mass or in vitro complex I activity, associated with a rearrangement of the cardiolipins and reduced integration of complex I into the electron transport chain supercomplexes. Strikingly, none of these defects were present in females. Interestingly, suppression of estradiol signaling by ovariectomy partially mimicked the male sensitivity to AMPK loss, notably the cardiac fibrosis and the rearrangement of cardiolipins, but not the cardiac function that remained protected.

**Conclusion:**

Our results confirm the close link between AMPK and cardiac mitochondrial function, but also highlight links with cardiac fibrosis. Importantly, we show that AMPK is differently involved in these processes in males and females, which may have clinical implications for the use of AMPK activators in the treatment of heart failure.

## Highlights

- AMPK is a metabolic sensor of cellular energy which regulates energy homeostasis.
- We generated a cardiac-specific inducible deletion of Ampkα2 and demonstrated that this deletion induces mild cardiac dysfunction in male only.
- Cardiac dysfunction observed in males was associated with cardiac fibrosis and cardiac cardiolipin remodeling that are not seen in females.
- Although no significant cardiac function alteration was noticed in ovariectomized female Ampkα2ciKO mice, these latter exhibited cardiac fibrosis and mild cardiolipins remodeling.
- Our results show a higher dependence on AMPK signaling fibrosis and cardiolipin biosynthesis/maturation in males, either due to the absence of female hormones protection or/and to the action of male hormones. This may contribute to the known difference in cardiovascular risk and outcome between sexes.

## Introduction

The AMP-activated protein kinase (AMPK) is a ubiquitous serine/threonine kinase which acts as a cellular “fuel gauge” regulating energy homeostasis [1]. This kinase is composed of a catalytic subunit (α) and two regulatory subunits (β and γ), each including several isoforms exhibiting differential tissue expression. In murine heart, the α-subunit type 2 (α2) is the major catalytic subunit isoform (70–80% of the total AMPK activity) and is mostly expressed in the cardiomyocytes, the α-subunit type 1 (α1) isoform being predominant in the non-myocyte cells [2, 3]. AMPK is activated under conditions of metabolic stress that deplete ATP [1] and modulate a number of physiological processes aiming at restoring energy balance [4]. Its activation requires allosteric stimulation by AMP and the α-subunit Thr172 phosphorylation by upstream kinases [5] such as Liver Kinase B1 (LKB1) [6] or Ca^2+^/calmodulin-dependent kinase kinase (CamKK) [7]. AMPK regulates energy metabolism by directly modulating the activity of key enzymes of cellular energetics and impacts expression of many genes involved in the energy production/consumption processes [8]. For instance, AMPK phosphorylates the peroxisome proliferator-activated receptor gamma co-activator 1α (PGC-1α), a master regulator of energy metabolism which, amongst others, stimulates mitochondrial biogenesis. It also enhances glucose and fatty acid uptake through the translocation of GLUT4 and CD36 transporters to the plasma membrane [9], boosts glycolysis by directly activating 6-phosphofructokinase 2 (PFK2) [10], and increases fatty acid oxidation by phosphorylating and inhibiting acetyl-CoA carboxylase (ACC) [11]. In a nutshell, AMPK stimulates catabolic processes producing ATP and inhibits anabolic processes consuming ATP [12].

Although the metabolic regulations of AMPK have been the main subject of a number of studies these last years, the role of AMPK extends beyond this energetic aspect. In the heart, it is known that this enzyme also exhibits non-metabolic effects like regulation of myocardial fibrosis and inhibition of cytosolic reactive oxygen species (ROS) production by NADPH oxidase [13–16]; this has to be considered when deciphering the part played by AMPK in cardiac physiology and pathophysiology. Our team has shown in a global and constitutive male *Ampkα2* knockout mouse model [17] that *Ampkα2* suppression led to significant decrease in mitochondrial oxidative capacities associated with a decrease in cardiolipin (CL) content suggesting a link between AMPK and the mitochondrial membrane components. Knowing the importance of mitochondrial phospholipid composition for suitable mitochondrial functions [18–20], a potential impact of AMPK activity on mitochondrial respiratory function through a regulation of CL biosynthesis/maturation could be proposed. Intriguingly, we also highlighted a concomitant decrease in AMPK activity and in CL content in cardiac left ventricle of male rats treated with doxorubicin [21]. In this study, doxorubicin treated female rats exhibited lesser alterations in cardiac function and mitochondrial oxidative capacities than males and showed preserved AMPK activity and CL content. Beyond the possible relationship between AMPK, CL and mitochondrial function, these interesting results could also evoke a possible role for AMPK in the sexual dimorphism extensively reported in cardiovascular diseases [22].

This last decade, AMPK has often been presented as a potential therapeutic target in many cardiovascular diseases since its pharmacological stimulation has been shown to be beneficial for the pathological heart under many circumstances such as ischemia/reperfusion, diabetic cardiomyopathy, pathological hypertrophy or heart failure (HF) [23–26]. ATP production deficiency has been extensively demonstrated in these pathologies [27] and, given the role of AMPK in energy metabolism, it is quite rational to consider the activation of AMPK as a plausible strategy to improve myocardial energetics in patients affected by these diseases. Yet, before going further in the development of such a therapy, a better understanding of AMPK role in cardiomyocyte homeostasis is required. The clarification of the connection between AMPK and CLs is of particular interest since our knowledge of this phenomenon is almost nonexistent in the one hand and, on the other hand the AMPK/CL axis could be part of the mechanisms responsible for the higher resistance of the female heart to cardiac diseases, at least before menopause.

Currently, inducible and tissue-specific deletion of a given protein is one of the most powerful tools to understand its cell autonomous roles in mature organs. Thus, we generated an original model of cardiac-specific *Ampkα2* knockout mouse inducible in adult by tamoxifen injection (1) to study the consequences of cardiomyocyte specific *Ampkα2* deletion on cardiac function, structure and energy metabolism; (2) to investigate the interplay between AMPK and cardiac CL and (3) to identify sex-specific AMPK-dependent regulations.

## Materials and methods

### Animals

*Ampkα2*^*floxΔE6/floxΔE6*^ (*AMPKα2*^*f/f*^) homozygous mice (kind gift of Dr. Viollet [28]) and α-MHC-MerCreMer (α-MHC-Cre) mice were crossed to create cardiac-specific and inducible knock-out (ciKO) mice (α-MHC-MerCreMer x *Ampkα2*^*floxΔE6/floxΔE6*^ (α-MHC-Cre/*Ampkα2*^*f/f*^)). Male and female α-MHC-Cre/*Ampkα2*^*f/f*^ mice were injected with tamoxifen (40 mg/kg i.p daily × 2 days) at the age of 8 weeks to induce *Ampkα2* deletion, thereby generating *Ampkα2* cardiac-specific inducible mice called *Ampkα2ciKO* or KO in this study. Littermate *Ampkα2*^*floxΔE6/floxΔE6*^ mice not carrying α-MHC-MerCreMer transgene were subjected to the same tamoxifen treatment and were used as control mice (*Ampkα2*^*f/f*^ or CT). Mice were euthanized 16 weeks after tamoxifen injection and hearts were rapidly excised, rinsed in cold calcium-free Krebs solution and weighed. A part of the left ventricle (LV) was immediately used for mitochondrial function assessment and another part was flash frozen in liquid nitrogen for further biochemical determinations. For ovariectomy, females had surgery at the age of 7 weeks (a week before tamoxifen injection). Anesthesia was induced by intraperitoneal injection of ketamine (50 mg/kg) and xylazine (8 mg/kg) and temperature during surgery was maintained at 37 °C using a heating pad. After incision in the pelvic region, ligation was performed between each uterine horn and ovary before excising both ovaries. Mice were sutured using absorbable suture silk (6-0) then placed under a heat lamp until they woke up. At awakening, mice were treated with buprenorphine (0.05 mg/kg, subcutaneous) to avoid post-surgery pain. All animal experimental procedures were approved by animal ethics committee of Paris-Saclay University, authorized by French government (Authorization Number: B9201901) and complied with directive 2010/63/EU of the European Parliament on the protection of animals used for scientific purposes.

### Echocardiography

Echocardiography was done using a 12-MHz transducer (Vivid 7, General Electric Healthcare) under 2.5% isoflurane gas anesthesia to assess cardiac function. The body temperature was maintained with a heating pad. M-mode echocardiography was used to determine left ventricular mass, fractional shortening and left ventricular ejection fraction.

### Histological analysis

Hearts were fixed in 4% paraformaldehyde, paraffin embedded and serially sectioned (5 μm).

Sections were stained with Sirius red. Fibrosis quantification was performed on 3–4 Sections (5–10 fields/section) per animal using Image J software.

### Mitochondrial functional assays in permeabilized cardiac fibers

Fibers prepared from the left ventricle were permeabilized with saponin as previously described [29] and kept on ice until use in a buffer containing in mM: 2.77 CaK_2_ ethyleneglycol tetraacetic acid (EGTA), 7.23 K_2_EGTA [100 nM free Ca^2+^], 6.56 MgCl_2_ [1 mM free Mg^2+^], 5.7 Na_2_ATP, 15 phosphocreatine, 20 taurine, 0.5 dithiothreitol (DTT), 50 K-methane sulfonate [160 mM ionic strength], 20 imidazole, pH 7.1. Measurements aiming at determining mitochondrial parameters were expressed per gram of dry fiber weight.

### Mitochondrial respiration

Mitochondrial respiratory function was studied in situ in saponin-permeabilized cardiac muscle fibers using a Clarke electrode as previously described [29]. A protocol was designed to measure oxygen consumption after successive addition of ADP (2 mM), malate (4 mM), l-glycerol-3-phosphate (4 mM), palmitoyl-CoA and carnitine (100 µM and 2 mM), pyruvate (1 mM), glutamate (10 mM), succinate (15 mM), amytal (an inhibitor of complex I, 1 mM) and the complex IV substrates N,N´,N´-tetramethyl-phenylenediamine dihydrochloride (TMPD)-ascorbate (0.5 mM–2 mM) (activator of complex IV) to a solution containing in mM: 2.77 CaK_2_ ethyleneglycol tetraacetic acid (EGTA), 7.23 K_2_EGTA [100 nM free Ca^2+^], 1.38 MgCl_2_, 3 K_2_HPO_4_, 20 taurine, 0.5 dithiothreitol (DTT), 90 K-methane sulfonate and 10 Na-methane sulfonate, 20 imidazole, pH 7.1 at 23 °C. Rates of respiration are given in µmoles O_2_/min/g dry weight.

### Enzyme activity

Frozen tissue samples were weighed, homogenized (Bertin Precellys 24) in ice-cold buffer (50 mg/ml) containing 4-(2-hydroxyethyl)-1-piperazineethanesulfonic acid (HEPES) 5 mM (pH 8.7), EGTA 1 mM, DTT 1 mM and 0.1% Triton X-100. Activities of citrate synthase (CS), cytochrome c oxidase (COX) and complex I were determined using standard spectrophotometric assays [30, 31].

### Immunoblotting

Frozen tissue samples were homogenized (Bertin Precellys 24) in ice-cold buffer containing HEPES 50 mM, KCl 50 mM, ethylenediaminetetraacetic acid (EDTA) 1 mM, β-glycerophosphate 5 mM, Triton X-100 0.1%, orthovanadate 1 mM, dithiothreitol 1 mM, sodium fluoride 50 mM, Na pyrophosphate 5 mM, phenylmethylsulfonyl fluoride 0.2 mM and antiprotease cocktail set (Calbiochem 539,134). Protein extracts were separated on SDS–polyacrylamide gel (8 to 12%) and then transferred to polyvinylidene difluoride membranes for Western blot. After an hour of blocking in PBS containing Tween 20 (0.1%) and non-fat milk (5%), the membranes were incubated overnight at 4 °C with primary antibody (Table 1). After washing, the membranes were incubated with a secondary antibody coupled with horseradish peroxidase for 1 h at room temperature and visualized using chemiluminescent substrate (Luminata™ Western Chemiluminescent HRP Substrates, Millipore). Light emission was detected by autoradiography and quantified using an image-analysis system (iBright FL1000, Invitrogen).

**Table 1:** Antibodies

Antibody	Supplier	Reference	Dilution
Actin	Santa Cruz	sc47778	1/10000
ACC	Cell Signaling	3676	1/1000
Phospho ACC	Cell Signaling	3661	1/1000
AMPKα1	Santa Cruz	sc19126	1/1000
AMPKα2	Santa Cruz	Sc19129	1/1000
Phospho AMPK	Cell signaling	2531	1/500
AMPK total	Cell signaling	2532	1/500
CS	Abcam	Ab96600	1/1000
mi-CK			
Grim19 (NDUFA13)	Abcam	ab110240	1/1000
Oxphos	Mitoscience	Ms604	1/500
Tubulin	Abcam	Ab7291	1/10000
Vinculin	Sigma	V9131	1/900

### Blue-native page

Mitochondria and mitochondrial electron transfer chain complexes were isolated from heart tissue using digitonin 5% (from Native Page Sample Prep Kit, Invitrogen) [32]. Fifty micrograms of proteins were loaded on a 3–16% acrylamide native gel (Invitrogen). After electrophoresis, proteins were transferred to a PVDF membrane. Immunoblotting was performed with Grim-19 antibody (Table 1) raised against NDUFA13 complex I subunit.

### Real-time quantitative RT-PCR analysis

Frozen tissue samples were weighed and homogenized (Bertin Precellys 24) in ice-cold buffer. Total ventricular RNA was extracted using standard procedure with Trizol reagent (Invitrogen). cDNAs were synthesized from 2 μg total RNA according to the protocol provided with the High Capacity cDNA Reverse Transcription Kit (Applied Biosystems, France). Real-time PCR was performed using TaqMan Low Density Array (TLDA) technology as previously described [33]. Pre-designed TaqMan probe and primer sets for target genes were chosen from an on-line catalog (Applied Biosystems, France). TLDA were designed to amplify 26 cDNA for each sample as follows: 21 target genes involved in energy metabolism, mitochondrial function and cardiac remodeling and 5 housekeeping genes (Table 2). Each PCR reaction was performed on 4 ng of cDNA in a volume of 1 µl. The thermal cycling conditions were 2 min at 50 °C and 10 min at 95 °C, followed by 40 cycles of 15 s at 95 °C and 1 min at 60 °C. Quantification was achieved using the ΔΔC_t_ method. The average C_t_ obtained in CT group was used as a calibrator and the geometric mean of the 5 housekeeping genes was used as the reference for normalization. The amplification efficiency of each probe is estimated maximal as described by the manufacturer.

**Table 2:** Primers

Gene name	Gene symbol	Function	Identification number (applied biosystems)
Natriuretic peptide type B	Nppb	Hypertrophy	Mm01255770_g1
Peroxisome proliferator-activated receptor, gamma, coactivator 1 alpha	Ppargc1a	Mitochondrial biogenesis	Mm00447181_m1
Peroxisome proliferator-activated receptor, gamma, coactivator 1 beta	Ppargc1b	Mitochondrial biogenesis	Mm00504730_m1
GA repeat binding protein, alpha	Gabpa	Mitochondrial biogenesis	Mm00484598_m1
Estrogen related receptor, alpha	Esrra	Mitochondrial biogenesis	Mm00433143_m1
Peroxisome proliferator-activated receptor alpha	Ppara	Mitochondrial biogenesis	Mm00440939_m1
Transcription factor A, mitochondrial	Tfam	Mitochondrial biogenesis	Mm00447485_m1
Mitochondrially encoded cytochrome c oxidase I	COX1	Mitochondrial biogenesis	Mm04225243_g1
Cytochrome c oxidase subunit IV isoform 1	Cox4i1	Mitochondrial biogenesis	Mm01250094_m1
Optic atrophy 1	Opa1	Mitochondrial dynamics	Mm00453879_m1
Mitofusin 1	Mfn1	Mitochondrial dynamics	Mm00612599_m1
Mitofusin 2	Mfn2	Mitochondrial dynamics	Mm00500120_m1
Dynamin 1-like	Dnm1l	Mitochondrial dynamics	Mm01342903_m1
Superoxide dismutase 2, mitochondrial	Sod2	Oxidative stress	Mm01313000_m1
Catalase	Cat	Oxidative stress	Mm00437992_m1
Glutathione peroxidase 1	Gpx1	Oxidative stress	Mm00656767_g1
Acyl-CoA synthetase long-chain family member 1	Acsl1	Cardiolipins biosynthesis	Mm00484217_m1
CDP-diacylglycerol synthase 1	Cds1	Cardiolipins biosynthesis	Mm01208328_m1
Cardiolipin synthase 1	Crls1	Cardiolipins biosynthesis	Mm00503002_m1
Tafazzin	Taz	Cardiolipins biosynthesis	Mm00504978_m1
Elongation of very long chain fatty acids-like 2	Elovl2	Cardiolipins biosynthesis	Mm00517086_m1
ELOVL family member 5, elongation of long chain fatty acids	Elovl5	Cardiolipins biosynthesis	Mm00506717_m1
Fatty acid desaturase 1 (delta 5 desaturase)	Fads1	Cardiolipins biosynthesis	Mm00507605_m1
Fatty acid desaturase 2 (delta 6 desaturase)	Fads2	Cardiolipins biosynthesis	Mm00517221_m1
Beta-2 microglobulin	B2m	Reference genes	Mm00437764_m1
Tyrosine 3-monooxygenase/tryptophan 5-monooxygenase activation protein, zeta polypeptide	Ywhaz	Reference genes	Mm03950126_s1
Ribosomal protein, large P2	Rplp2	Reference genes	Mm00782638_s1
Polymerase (RNA) II (DNA directed) polypeptide A	Polr2a	Reference genes	Mm00839493_m1
	18S	Reference genes	Hs99999901_s1

Quantification of *Col1a1* (forward 5'-CTCAAGATGTGCCACTCTGACT-3', reverse 5'-CTCCATGTTGCAGTAGACCTTG-3'), and *Col3a1* (5'-GAT GGAAACCCTGGATCAGA -3', 5'-GCACCAGGAGAACCATTTTC-3') mRNA were assessed using the SYBR®Green method on a LightCycler rapid thermal cycler (Roche Diagnostics) as previously described [34]. For each target gene, a standard curve was constructed from the analysis of a fivefold cDNA serial dilution and used for samples concentration calculation. Ywhaz (5'-AGACGGAAGGTGCTGAGAAA-3', 5'-GAAGCATTGGGGATCAAGAA-3') was used as housekeeping gene as its expression did not differ between groups.

### Electron microscopy

Left ventricular papillary muscles were isolated from three control and three KO mouse hearts, fixed with 2% glutaraldehyde in cacodylate buffer (in mM: 150 Na-cacodylate, 2CaCl2, pH 7.3) for 1 h, post-fixated by 1% osmium tetroxide in cacodylate buffer for 30 min and stained with 1% aqueous solution of uranyl acetate. After dehydration in graded ethanol series and acetone, the tissue was embedded in Durcupan (ACM Fluka). Ultrathin (58–60 nm) longitudinal sections were cut using an ultramicro-tome (Power-Tome MT-XL, RMC/Sorvall, Tucson, AZ, USA). The sections were mounted on formvar-coated copper grids, contrasted with lead citrate and examined with a JEM 1200 electron microscope (Jeol, Tokyo, Japan) at 80 kV. Random images of cardiomyocytes were recorded with a CCD camera (Gatan DualVision 300 W) at a magnification of 15,000 and analyzed using Graphic Cell Analyzer14.

### Cardiolipin content

Cardiolipin analysis was performed by liquid chromatography using corona-CAD detector as previously described [21]. As cardiolipins are almost exclusively located in the inner mitochondrial membrane, lipids were extracted from 10 to 20 mg of heart homogenized in PBS using the Folch method [35]. Total lipids were extracted by adding 1.5 mL of methanol and 3 mL of chloroform to the tissue suspension. After centrifugation at 1000 g for 10 min, the lower phase containing total lipids was collected and evaporated to dryness at room temperature under nitrogen gas. The samples were resuspended in 100 μl of chloroform per 10 mg of heart and subsequently analyzed. Quantification of cardiolipins was performed on a Dionex U-3000 RSLC system (ThermoFisher Scientific) equipped with a Corona-CAD Ultra (ThermoFisher Scientific). Separation of lipids was performed with a PVA-Sil column (150 × 2.1 mm I.D., 120 A) (YMC Europe GmbH) at 35 °C. Chromatographic method was inspired from the method developed by Imbert et al*.* [36]. The flow rate was set at 0.400 mL/min and 5 μl of sample were injected. The corona-CAD nebulizer was set at 30 °C and the nitrogen pressure was set at 5 bars. Standard curve from 0.5 to 0.025 mg/mL of cardiolipin sodium salt from bovine heart (98% purity from Sigma-Aldrich) was used.

### Statistical analysis

All results are expressed as mean ± SEM. To assess significance, we performed Student’s t test when the experimental design compared only 2 groups or two-way ANOVA for independent factors when appropriate for the experimental design; Tukey post hoc tests were used to identify significant differences between means. Results were considered statistically different when p-value was strictly lower than 0.05. Symbols referring to statistics are defined in each figure legend.

## Results

### Cardiac-specific tamoxifen-induced loss of Ampkα2 in male and female mice

**Fig. 1:**
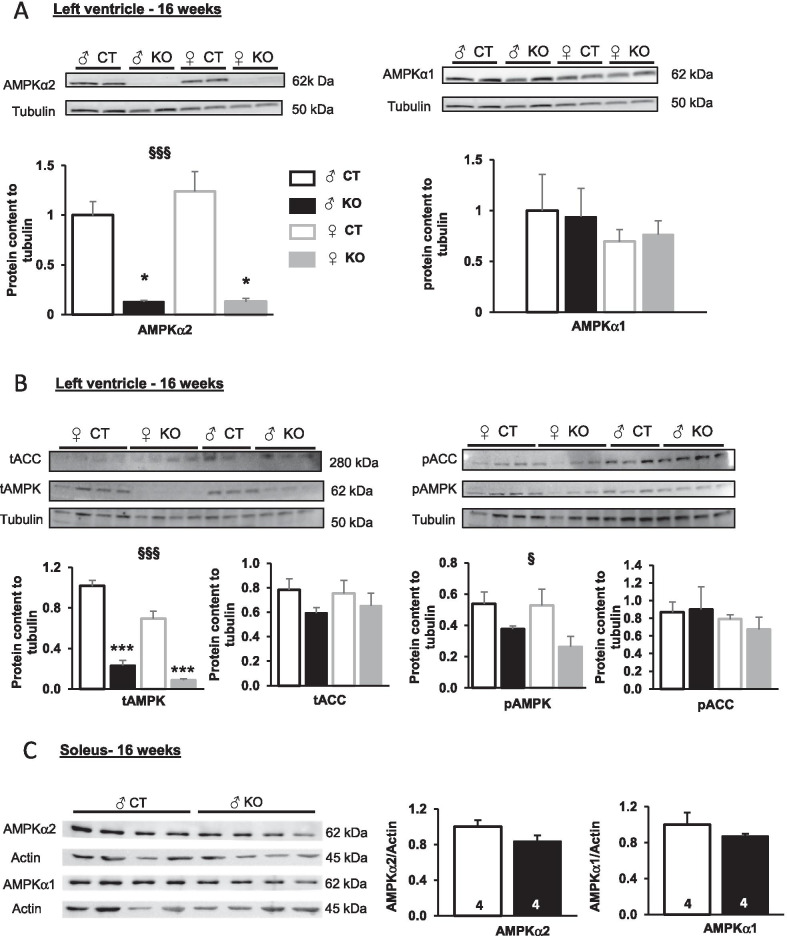
Cardiac-specific *Ampkα2* inactivation 16 weeks after tamoxifen injection in adult male and female mice. **A** Protein content of AMPKα2 and AMPKα1 in left ventricle (LV) homogenates. Tubulin is used as a loading control. **B** Immunoblotting of total AMPK (tAMPK), total ACC (tACC), phosphorylated-AMPK (pAMPK) and phosphorylated-ACC (pACC) in LV. Tubulin is used as a loading control. **C** Protein content of AMPKα2 and AMPKα1 in skeletal muscle. Actin is used as a loading control. (*n* = 3 to 4 per experimental group). ANOVA: §*p* ≤ 0.05, §§*p* ≤ 0.01, §§§*p* ≤ 0.001 for the genotype effect. Post hoc Tukey test: *p < 0.05, ***p < 0.001 *Ampkα2*^*f/f*^* vs Ampkα2ciKO*

At 8 weeks of age, α-MHC-Cre/*Ampkα2*^*f/f*^ mice were given tamoxifen to induce exon 6 excision from the floxed *Ampkα2* alleles. Sixteen weeks after *Ampkα2* deletion, AMPKα2 protein level in left ventricle (LV) homogenates exhibited reductions of 95 ± 1% in *Ampkα2ciKO* males and 90 ± 3% in females when compared with the respective control *Ampkα2*^*f/f*^ mice (Fig. 1A). This drastic decrease in AMPKα2 content was not compensated by any change in AMPKα1 expression (Fig. 1A) and led to an important decrease in total AMPKα content in the LV of both sexes (Fig. 1B). The antibody recognizing both isoforms showed up to at least 75% reduction of signal in the *Ampkα2ciKO* heart (Fig. 1B, total AMPK), further demonstrating the predominant expression of AMPKα2 over AMPKα1 when considering the whole myocardium. As AMPKα2 is depleted over 95%, we calculated that AMPKα1 accounts for 80% of the remnant total AMPK signal in the *Ampkα2ciKO* heart. Whether in males or females, the important decrease in AMPKα2 content was associated with a lower amount of phosphorylated AMPKα (Thr 172), the active form of AMPK. The antibody recognizing both phosphorylated AMPKα1 (Thr 183), and AMPKα2 (Thr 172) showed a 30% reduction of signal. Thus, the remnant 70% phospho-AMPK signal comes most probably mostly from phosphorylated AMPKα1. Even though this set of data could suggest a decrease in AMPK activity, phosphorylation of acetyl-CoA carboxylase (ACC), a target of AMPKα1/AMPKα2, was not significantly different between all groups while the total amount of ACC was unchanged in *Ampkα2ciKO* mice (Fig. 1B). The cardiac specificity of *Ampkα2* deletion in the present animal model was confirmed by the similar protein level of AMPKα2 and AMPKα1 in skeletal muscles of *Ampkα2ciKO* and *Ampkα2*^*f/f*^ male mice (Fig. 1C).

### Ampkα2ciKO male mutants develop a mild left ventricular systolic dysfunction

**Fig. 2:**
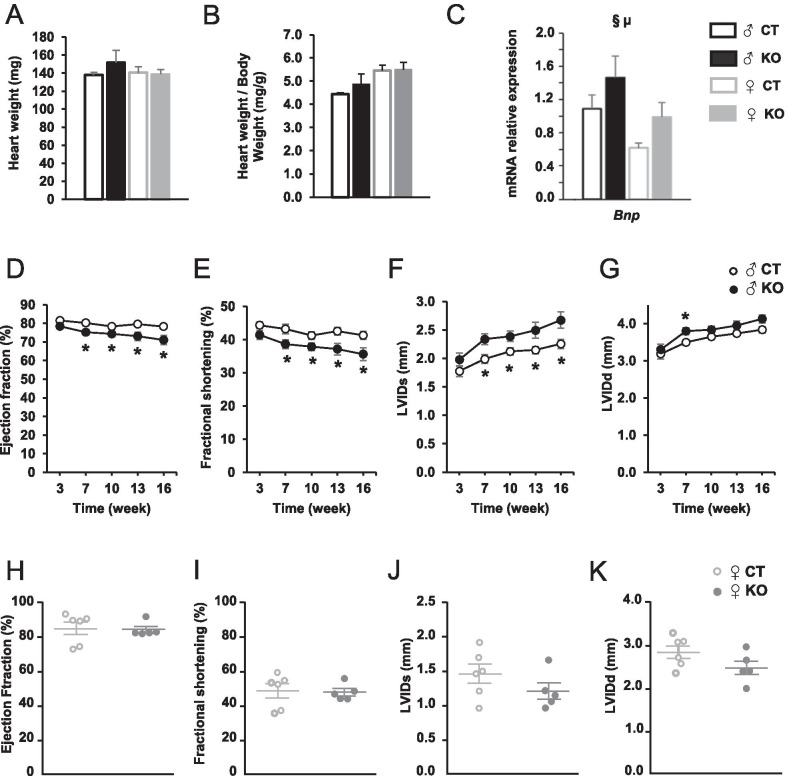
Progression of cardiac function during 16 weeks after cardiac-specific *Ampkα2* deletion in male and female mice. Heart weight (**A**), heart weight-to-body weight ratio (**B**), and brain natriuretic peptide (*Bnp*) gene expression (**C**) in left ventricle after 16 weeks of *Ampkα2* deletion. Progression of left ventricular ejection fraction (**D**), progression of left ventricular fractional shortening (**E**), progression of left ventricular internal dimension at end-systole (LVIDs) (**F**) and progression of left ventricular internal dimension at end-diastole (LVIDd) (**G**) in males. Left ventricular ejection fraction (**H**), left ventricular fractional shortening (**I**), left ventricular internal dimension at end-systole (LVIDs) (**J**) and left ventricular internal dimension at end-diastolic (LVIDd) (**K**) after 16 weeks of *Ampkα2* deletion in females. (*n* = 5 to 7 per experimental group). ANOVA: §*p* ≤ 0.05 for the genotype effect; µ *p* ≤ 0.05 for the sex effect. Post hoc Tukey test: *p < 0.05 *Ampkα2*^*f/f*^* vs Ampkα2ciKO* (same sex)

Sixteen weeks after *Ampkα2* deletion, mutant mice did not display any change in heart weight as judged by absolute organ weight and heart weight-to-body weight ratio (Fig. 2A and B). The expression of the gene encoding brain natriuretic peptide (*Bnp*) was significantly increased after *Ampkα2* deletion and was significantly lower in females than in males (Fig. 2C). Cardiac function of *Ampkα2ciKO* mice and their control littermates was assessed by serial echocardiography 3, 7, 10, 13 and 16 weeks after the first tamoxifen injection in males and by a single echocardiography at 16 weeks in females. In males, while echocardiography parameters did not show any difference between control and mutant mice 3 weeks after induction of *Ampkα2* deletion, significant decreases in LV ejection fraction (EF) and fractional shortening (FS) as well as a significant increase in end-systolic left ventricular internal diameter (LVIDs) were observed starting 7 weeks after tamoxifen treatment (Fig. 2D–G and Table 3). However, these alterations of cardiac systolic function were still slight after 16 weeks and no significant impact on cardiac output was noticed at this time point (Table 3). In these animals, diastolic function seemed to be more preserved since most of the diastolic echocardiography parameters were similar in CT and *Ampkα2ciKO* mice at 16 weeks. Nevertheless, left ventricular end-diastolic volume was significantly increased in *Ampkα2* deleted male mice at this time point (Table 3) and LVIDd tended to be higher in this group, even showing a significant difference with *Ampkα2*^*f/f*^ at 7 weeks (Fig. 2G). In females, echocardiography did not reveal any significant modifications between CT and KO in LV ejection fraction (Fig. 2H), fractional shortening (Fig. 2I) and ventricular internal diameters (Fig. 2J–K and Table 4). This is in contrast with the mild left ventricular systolic dysfunction observed in KO male mice and suggests that female heart could be more resistant to *Ampkα2* deletion.

**Table 3:** Echocardiographic parameters in males 16 weeks after induction of *Ampkα2* deletion by tamoxifen injection

Echocardiographic parameters	*Ampkα2*^*f/f*^* n* = 7	*Ampkα2ciKO n* = 7
HR (beats/min)	519 ± 42	533 ± 47
IVSd (mm)	0.781 ± 0,158	0.831 ± 0.148
IVSs (mm)	1.276 ± 0.191	1.330 ± 0.191
LVIDd (mm)	3.84 ± 0.30	4.13 ± 0.38
LVIDs (mm)	2.26 ± 0.28	2.67 ± 0.49*
LVPWd (mm)	0.738 ± 0.219	0.741 ± 0.192
LVPWs (mm)	1.286 ± 0.153	1.196 ± 0.181
EDV (ml)	0.14 ± 0.03	0.18 ± 0.05*
ESV (ml)	0.03 ± 0.01	0.06 ± 0.04*
EF (%)	78.3 ± 4.53	71.1 ± 8.61*
SV (ml)	0.11 ± 0.02	0.12 ± 0.02
FS (%)	41.32 ± 4.10	35.62 ± 6.48*
CO (ml/min)	58.3 ± 12.5	65.9 ± 11.7

Statistical differences are highlighted in bold

HR, heart rate; IVSd, interventricular septal thickness at end-diastole; IVSs, interventricular septal thickness at end-systole; LVIDd, left ventricular internal dimension at end-diastole; LVIDs, left ventricular internal dimension at end-systole; LVWPd, left ventricular posterior wall thickness at end-diastole; LVWPs, left ventricular posterior wall thickness at end-systole; EDV, end-diastolic volume; ESV, end-systolic volume; EF, left ventricular ejection fraction; SV, stroke volume; FS, left ventricular fractional shortening; CO, cardiac output; *p < 0.05 *Ampkα2*^*f/f*^ vs* Ampkα2ciKO*

**Table 4:** Echocardiographic parameters in females 16 weeks after induction of *Ampkα2* deletion by tamoxifen injection

Echocardiographic parameters	*Ampkα2*^*f/f*^* n* = 7	*Ampkα2ciKO n* = 6
HR (beats/min)	384 ± 30	447 ± 35
IVSd (mm)	0.97 ± 0.12	0.99 ± 0.09
IVSs (mm)	1.49 ± 0.12	1.56 ± 0.15
LVIDd (mm)	2.76 ± 0.21	2.55 ± 0.16
LVIDs (mm)	1.50 ± 0.16	1.30 ± 0.12
LVPWd (mm)	1.10 ± 0.07	1.03 ± 0.03
LVPWs (mm)	1.48 ± 0.08	1.46 ± 0.10
EDV (ml)	0.07 ± 0.01	0.05 ± 0.01
ESV (ml)	0.12 ± 0.02	0.09 ± 0.01
EF (%)	81.1 ± 4.4	82.4 ± 0.4
SV (ml)	0.05 ± 0.01	0.05 ± 0.01
FS (%)	45.4 ± 4.7	47.1 ± 1.0
CO (ml/min)	18.4 ± 4.3	21.2 ± 3.6

HR, heart rate; IVSd, interventricular septal thickness at end-diastole; IVSs, interventricular septal thickness at end-systole; LVIDd, left ventricular internal dimension at end-diastole; LVIDs, left ventricular internal dimension at end-systole; LVWPd, left ventricular posterior wall thickness at end-diastole; LVWPs, left ventricular posterior wall thickness at end-systole; EDV, end-diastolic volume; ESV, end-systolic volume; EF, left ventricular ejection fraction; SV, stroke volume; FS, left ventricular fractional shortening; CO, cardiac output

### Cardiac-specific Ampkα2 deletion is associated with ventricular fibrosis and mitochondrial alterations in males

**Fig. 3:**
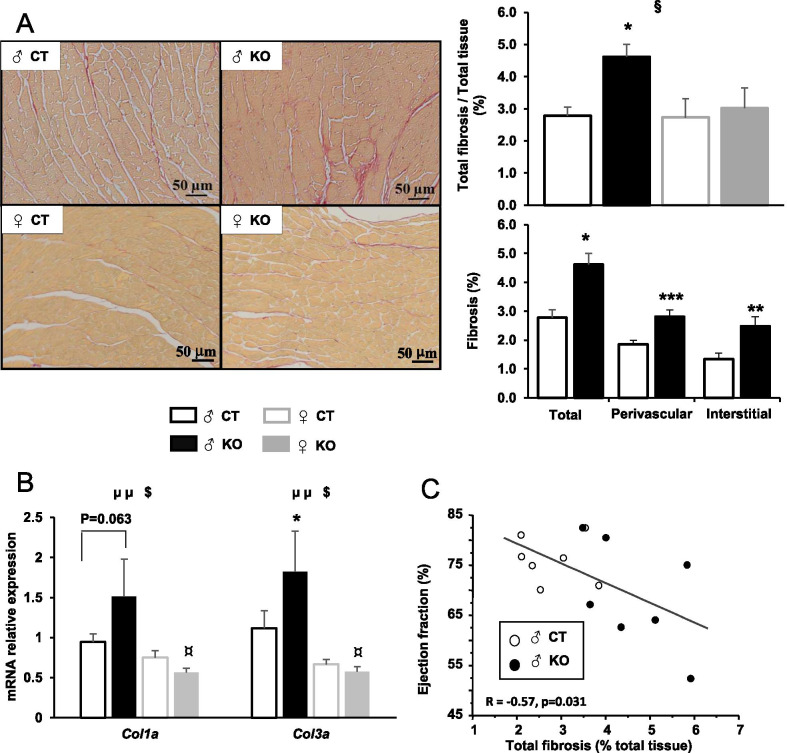
Myocardial fibrosis 16 weeks after cardiac-specific *Ampkα2* deletion in male and female mice. **A** Representative pictures of fibrosis analysis by Sirius red staining of subequatorial heart section (right panels), proportion of total fibrosis in males and females (upper right panel) and % of total, perivascular and interstitial fibrosis in males (lower right panel). **B** Left ventricular mRNA expression level of collagen 1 (*Col1a1*) and collagen 3 (*Col3a1*). **C** Correlation curve between ejection fraction and total fibrosis in males. (*n* = 5 to 7 per experimental group). ANOVA: §*p* < 0.05 for the genotype effect; µµ *p* ≤ 0.01 for the sex effect; $*p* < 0.05 for interaction; Post hoc Tukey test: *p < 0.05, **p < 0.01, ***p < 0.001 *Ampkα2k*^*f/f*^* vs Ampkα2ciKO* (same sex); ¤*p* < 0.05 males *vs* females (same genotype)

Inasmuch as ventricular dysfunction has been reported to be often associated with fibrosis which can change tissue properties and negatively affect contraction and/or relaxation, the consequences of *Ampkα2* deletion on fibrosis have been assessed using Sirius red staining. In *Ampkα2ciKO* males, after 16 weeks of deletion, total fibrosis of LV was markedly increased and collagen deposition was observed in the vicinity of the vessels (perivascular fibrosis) as well as around the cardiomyocytes (interstitial fibrosis) (Fig. 3A). No cardiac fibrosis was observed in female KO mice compared to CT mice. In line with these observations, the expression level of *Col3a1* was significantly increased and *Col1a1* expression tended to be higher in mutant males in comparison with CT males while CT and KO female mice exhibited similar expression level of these genes (Fig. 3B). Interestingly, a significant correlation between EF and percentage of total fibrosis has been highlighted in males (Fig. 3C).

**Fig. 4:**
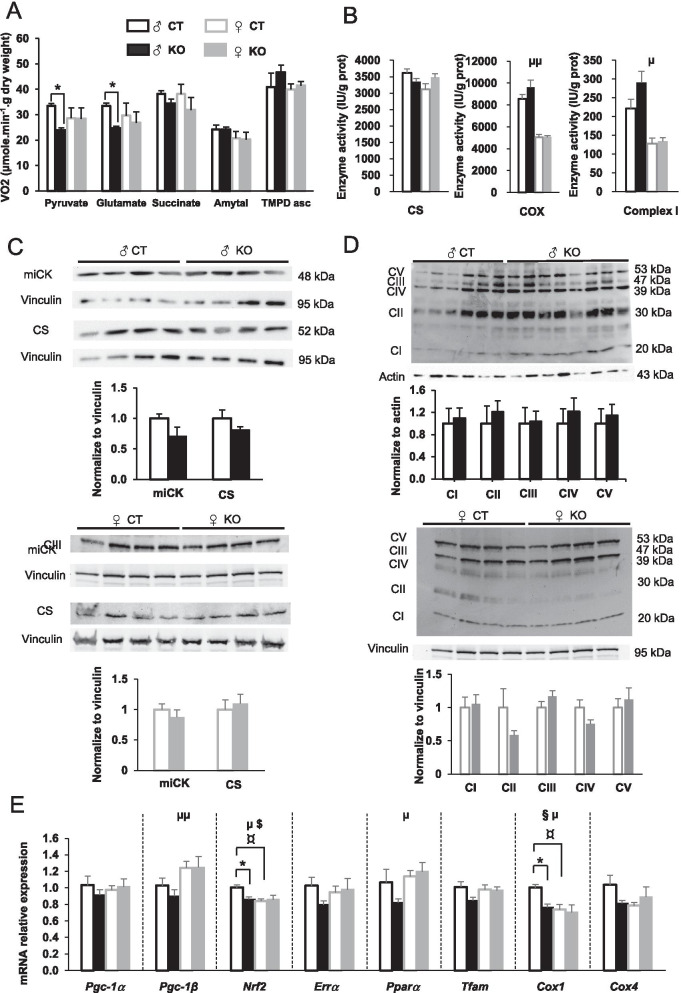
Left ventricle mitochondrial phenotype 16 weeks after cardiac-specific *Ampkα2* deletion in male and female mice. **A** Rate of respiration after successive addition of pyruvate (1 mM), glutamate (10 mM), succinate (15 mM), amytal (1 mM) and TMPD-ascorbate (0.5:2 mM). **B** Citrate synthase (CS), cytochrome c oxidase (COX) and complex I enzymatic activities. **C** Immunoblotting of citrate synthase (CS) and mitochondrial creatine kinase (miCK). Vinculin is used as a loading control. **D** Total protein content of 5 subunits of oxidative phosphorylation complexes: C-I-20 (complex I), C-II-30 (complex II), C-III-Core 2 (complex III), C-IV-COXI (complex IV) and C-V-α (complex V). **E** mRNA expression level of genes related to mitochondrial biogenesis: *Pgc-1α*, *Pgc-1β*, *Nrf2 (gabpa)*, *Errα*, *Pparα*, *Tfam*, *Cox1* and *Cox4*. (*n* = 5 to 7 per experimental group). ANOVA: §*p* < 0.05 for the genotype effect; µ *p* < 0.05, µµ *p* < 0.01, µµµ *p* < 0.001 for the sex effect; $*p *< 0.05 for interaction; Post hoc Tukey test: *p < 0.05 *Ampkα2*^*f/f*^ vs* Ampkα2ciKO* (same sex); ¤*p* < 0.05, ¤¤*p* < 0.01 males vs females (same genotype)

The loss of AMPKα2 negatively impacted mitochondrial function only in males since respiration rates measured in permeabilized fibers in presence of malate, pyruvate and glutamate were largely decreased in *Ampkα2ciKO* male mice (Fig. 4A), whereas these parameters were similar to controls in female *Ampkα2ciKO* mice (Fig. 4A). In males, the subsequent addition of succinate with or without amytal (inhibitor of complex I) led to the normalization of mitochondrial O_2_ consumption to control values in mutant mice suggesting that complex II activity can compensate for the deficit in complex I-respiration in the context of saturating succinate concentration (Fig. 4A). This indicates that maximal mitochondrial oxidative capacities were comparable in both male groups, what was confirmed by the equivalent TMPD-stimulated cytochrome C-dependent respiration rate (Fig. 4A) and the similar activity of cytochrome oxidase (COX) and citrate synthase (CS) (Fig. 4B), the latter being traditionally used as a marker of mitochondrial mass. In line with enzyme activity, no significant difference in CS protein level was noticed in male mutant mice compared to *Ampkα2*^*f/f*^ mice either (Fig. 4C). Altogether, these results suggest that the decrease in mitochondrial respiration observed in males when malate, pyruvate and glutamate were added in the respiration chamber would be due to alterations of complex I of electron transfer chain (ETC). Yet, activity of complex I measured on protein homogenate of LV and the protein content of C-I-20 (a complex I subunit) were not changed by the loss of AMPKα2 (Fig. 4B and D). Incidentally, none of the subunits of the five complexes of ETC showed alterations 16 weeks after *Ampkα2* deletion in males (Fig. 4D). Regarding females, although COX and complex I activities were significantly lower than in males (regardless genotype), no enzyme activity (CS, COX, complex I) or mitochondrial protein expression (CS and subunits of the ETC complexes) measured in this study were altered by 16 weeks of *Ampkα2* deletion (Fig. 4B–D).

Despite the fact that mitochondrial mass and maximal oxidative capacities seemed to be similar in both male groups, the significant decrease in *Nrf2* and *Cox1* expression and the trend towards lower expression of *Errα*, *Tfam, Pparα* and *Cox4* displayed by male mutant mice in comparison with controls could suggest a slight alteration of mitochondrial biogenesis while *Pgc-1α* and *Pgc-1β* expression seemed not to be affected by the loss of AMPKα2 (Fig. 4E). Expression of all these genes was similar to control after 16 weeks of *Ampkα2* deletion in females (Fig. 4E). Note that the expression of *Pgc-1β*, *Nrf2, Pparα* and *Cox1* showed clear sexual dimorphism (Fig. 4E).

**Fig. 5:**
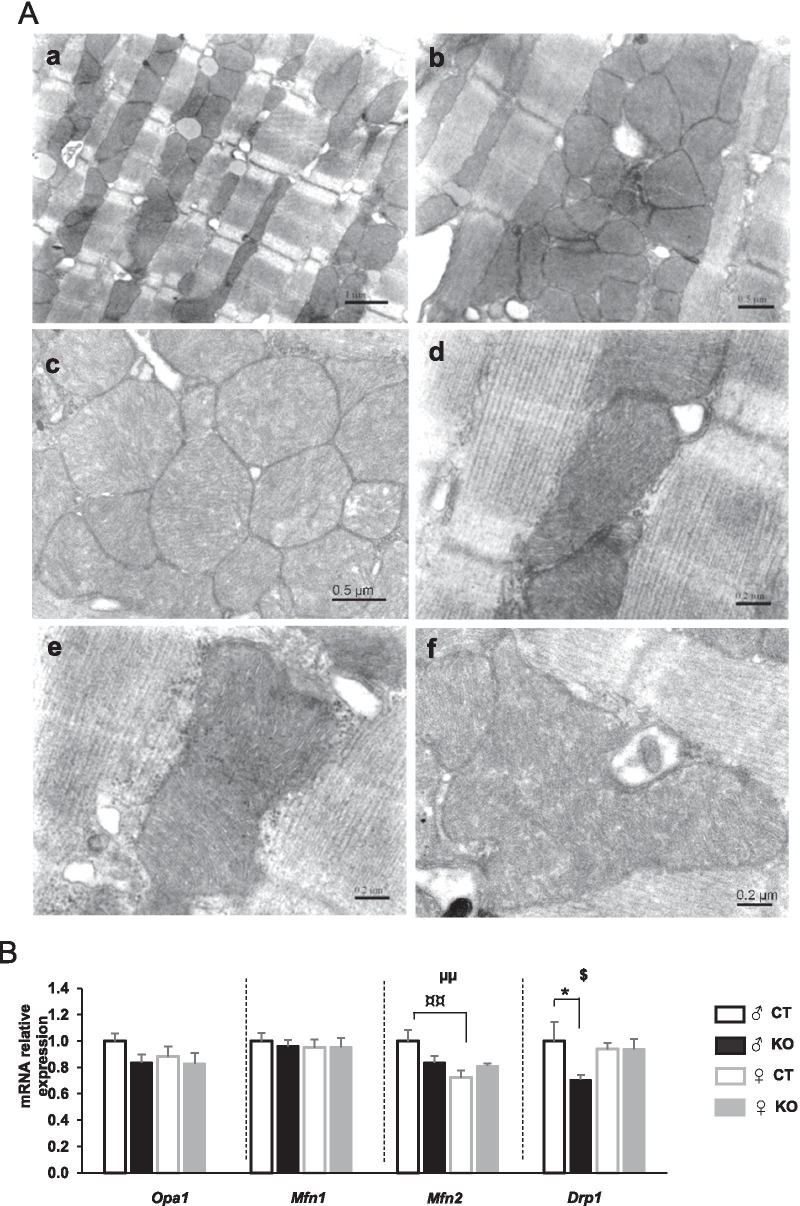
Cardiomyocyte cytoarchitecture and mitochondrial morphology 16 weeks after cardiac-specific *Ampkα2* deletion in male mice. **A** Transmission electron micrograph of left ventricle of *Ampkα2*^*f/f*^ (Aa) and *Ampkα2ciKO* (Ab-Af) mice. **B** mRNA expression level of genes encoding mitochondrial dynamics proteins: optic atrophy protein 1 (Opa1), Mitofusin 1 and 2 (Mfn1 and Mfn2) and dynamin-related protein 1 (Drp1). ANOVA: µµ *p* < 0.01 for the sex effect; $ *p* < 0.05 for interaction; Post hoc Tukey test: * *p* < 0.05 *Ampkα2*^*f/f*^ vs* AmpkciKO* (same sex); ¤¤*p* < 0.01 males *vs* females (same genotype)

As cardiac function and energy metabolism are strongly related to cardiomyocytes ultrastructure, cytoarchitecture analyses of cardiomyocytes have been thoroughly done using TEM. This revealed that, beyond mitochondrial biogenesis, mitochondrial life cycle could be more largely disturbed by *Ampkα2* deletion. Indeed, in LV of male *Ampkα2ciKO* mice, mitochondria were not as well organized along the myofilaments as in controls (Fig. 5Aa and Ab), exhibited a marked heterogeneity in size (Fig. 5Ab and Ac) and were frequently organized in clusters (Fig. 5Ac). Important disorganization of cristae (Fig. 5Ad and Ae) and “giant” mitochondria (Fig. 5Af) were also frequently noticed in male mutant mice. The latter point could be partly explained by a potential alteration of mitochondrial dynamics process since the expression of the pro-fission gene *Drp1* was lower when AMPKα2 was not expressed while the expressions of *Mfn1*, *Mfn2* and *Opa1*, involved in mitochondrial fusion, were not significantly changed by the deletion (Fig. 5B). Of note, no alteration in the expression of all these genes was noticed in females (Fig. 5B).

### Inner mitochondrial membrane is altered in male Ampkα2ciKO mice

**Fig. 6:**
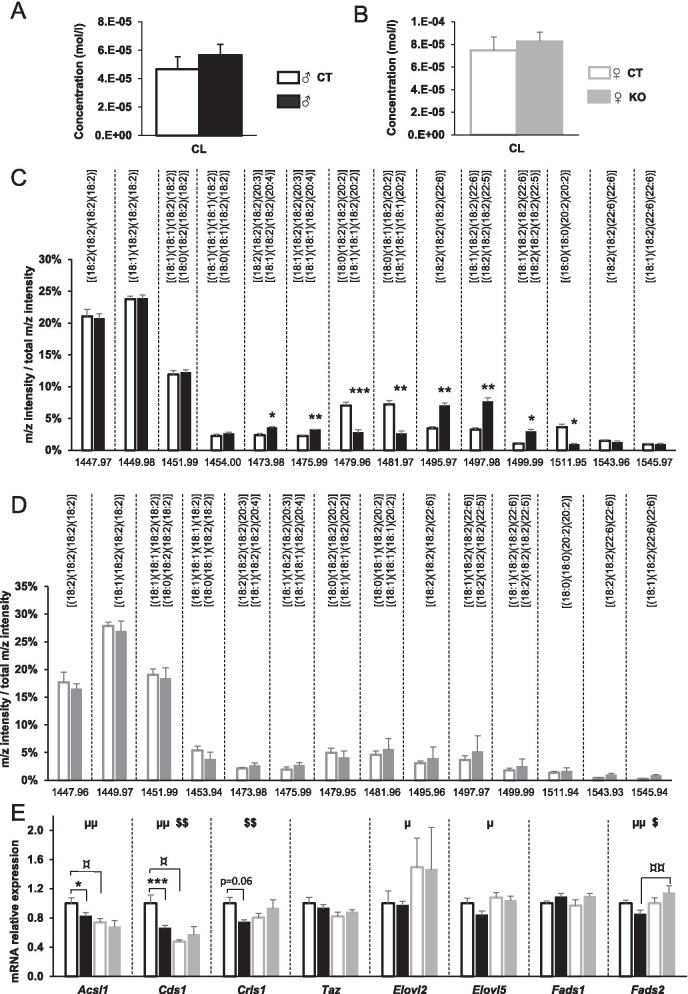
Left ventricular cardiolipin profile in male and female mice 16 weeks after cardiac-specific *Ampkα2* deletion. **A** Total cardiolipin content in male mice. **B** Total cardiolipin content in female mice. **C** Relative content of each identified cardiolipin species in male mice. **D** Relative content of each identified cardiolipin species in female mice. **E** Expression of genes related to cardiolipin biosynthesis and maturation: acyl-CoA synthetase long-chain family member 1 (Acsl1), CDP-diacylglycerol synthase 1 (Cds1), cardiolipin synthase 1 (Crls1), tafazzin (Taz), elongation of very long chain fatty acids-like 2 (Elovl2), elongation of long chain fatty acids (Elovl5), fatty acid desaturase 1 (Fads1) and fatty acid desaturase 2 (Fads2). ANOVA: µ *p* < 0.05, µµ *p* ≤ 0.01 for the sex effect; $*p* < 0.05, $$*p* < 0.01 for interaction; Post hoc Tukey test: * *p* < 0.05; **p < 0.01, ***p < 0.001 *Ampkα2*^*f/f*^* vs Ampkα2ciKO* (same sex); ¤*p* < 0.05, ¤¤*p* < 0.01 males *vs* females (same genotype)

**Fig. 7:**
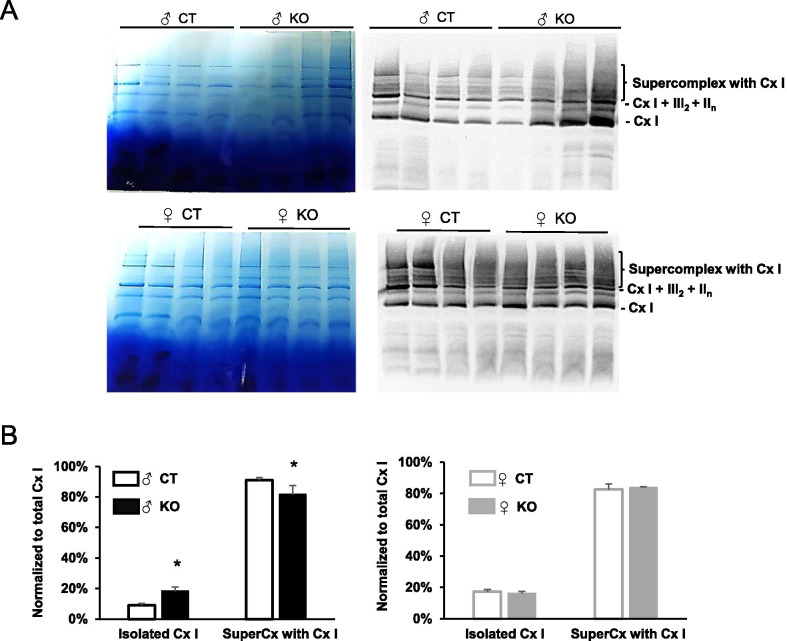
Left ventricular integration of electron transfer chain complex I within the mitochondrial supercomplexes 16 weeks after cardiac-specific *Ampkα2* deletion induction in male and female mice. **A** Mitochondrial supercomplexes were first separated by BN-PAGE (left panels), then transferred to PVDF membrane and incubated with complex I antibody (right panels) in CT and KO males (upper panels) and females (lower panels). **B** Quantification of isolated complex I and complex I including supercomplexes in males (left panel) and females (right panel). (*n* = 4 per experimental group). *p < 0.05 *Ampkα2*^*f/f*^ vs* Ampkα2ciKO* (same sex)

Whereas the alteration of complex I function 16 weeks after *Ampkα2* deletion was one of the most noticeable result, the apparent discrepancy between the respiration data indicating a complex I deficiency and the biochemical data showing normal complex I activity and protein content (subunit C-I-20) raised questions. We thus sought to determine whether the microenvironment surrounding complex I within the inner mitochondrial membrane could be different in mutant mice and could explain its lack of efficiency within the ETC. Sixteen weeks after tamoxifen injection, *Ampkα2ciKO* male mice clearly showed a marked rearrangement in the mitochondrial membrane cardiolipin (CL) profile (Fig. 6). Although the total CL content in KO was similar to the *Ampkα2*^*f/f*^ mice in both sexes (Fig. 6A and B), the amount of several CL species was affected by the loss of AMPKα2 in males only (Fig. 6C and D). The proportion of CL containing 20-carbon acyl chains was significantly changed in *Ampkα2ciKO* since a large reduction of CL with eicosadienoic acid (20:2) chains and a slight increase in CL containing eicosatrienoic acid (20:3) or eicosatetraenoic acid (20:4) were observed (Fig. 6C). Male KO mice also exhibited a much larger fraction of CL containing docosahexaenoic (22:6) or docosapentaenoic (22:5) acyl chains. Interestingly, these alterations in mitochondrial membrane composition were associated with significant decreases in expression of *Acsl1* and *Cds1* as well as a trend towards a lower expression of *Crls1* in males only (Fig. 6E), these three genes encoding important enzymes involved in CL synthesis. None of the enzymes involved in the production of CL was significantly impacted in the female *Ampkα2ciKO* mice 16 weeks after deletion when compared to female CT mice. Of note, the expression of many genes encoding enzymes involved in CL biosynthesis/maturation showed sexual dimorphism. As complexes of the respiratory chain are usually arranged in supercomplexes, we thought to investigate the assembly of complex I in these structures. In males, the CL remodeling was associated with a lower propensity of complex I to interact physically with the other complexes of ETC. Indeed, a lower proportion of complex I took part in the organization of mitochondrial supercomplexes and a higher part of complex I was found isolated; no such changes were revealed in female mice (Fig. 7A and B).

### Ovariectomy makes female mice more sensitive to the loss of AMPKα2

**Fig. 8:**
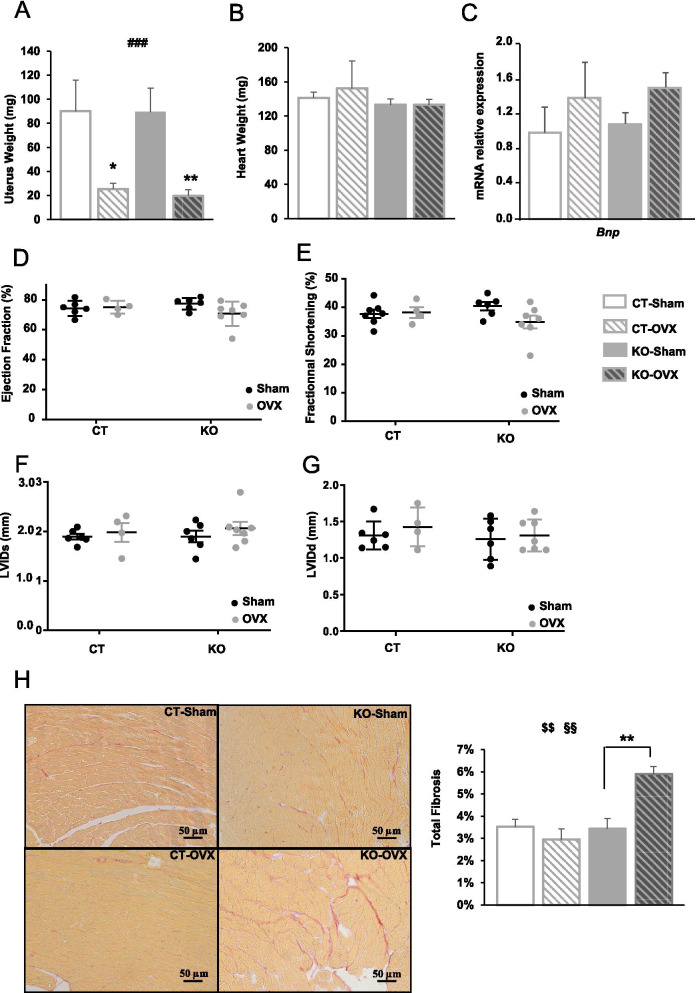
Cardiac function and myocardial fibrosis in control (Sham) and ovariectomized (OVX) female mice 16 weeks after cardiac-specific *Ampkα2* deletion. **A** Uterus weight. **B** Heart weight. **C** brain natriuretic peptide (*Bnp*) gene expression in left ventricle. **D** Left ventricular ejection fraction. **E** Left ventricular fractional shortening. **F** Left ventricular internal dimension at end-systolic (LVIDs). **G** Left ventricular internal dimension at end-diastolic (LVIDd). **H** Representative pictures of fibrosis analysis by Sirius red staining of subequatorial heart section. **I** Percentage of total fibrosis. ANOVA: # *p* < 0.05, ### *p* < 0.001 for the OVX effect; §§*p* < 0.01 for the genotype effect, $$*p* < 0.01 interaction. Post hoc Tukey test: *p < 0.05, **p < 0.01 Sham vs OVX

Obviously, one major difference between males and females lies in hormonal status which is incidentally known to be largely involved in the sexual dimorphism reported in cardiovascular diseases [22]. To investigate the potential interactions between AMPK and female hormones that might explain the differences observed between males and females in the present mouse model, ovariectomy (OVX) was performed in CT and *Ampkα2ciKO* mice. Seventeen weeks after ovariectomy, mice were sacrificed and the success of the surgery was confirmed by the large reduction of uterus weight in both OVX groups (Fig. 8A). Interestingly, although ablation of the ovaries did not induce clear modulations of heart weight, cardiac *Bnp* expression and heart function in *Ampkα2ciKO* mice (Fig. 8B–G), this surgery led to a significant increase in total fibrosis in *Ampkα2ciKO* mice only (Fig. 8H–I). In this group, total fibrosis reached 5.9 ± 0.3% of the tissue (Fig. 8I), so a level like the one observed in the male *Ampkα2ciKO* mice in which total fibrosis reached 4.6 ± 0.4% of the tissue (Fig. 3A).

**Fig. 9:**
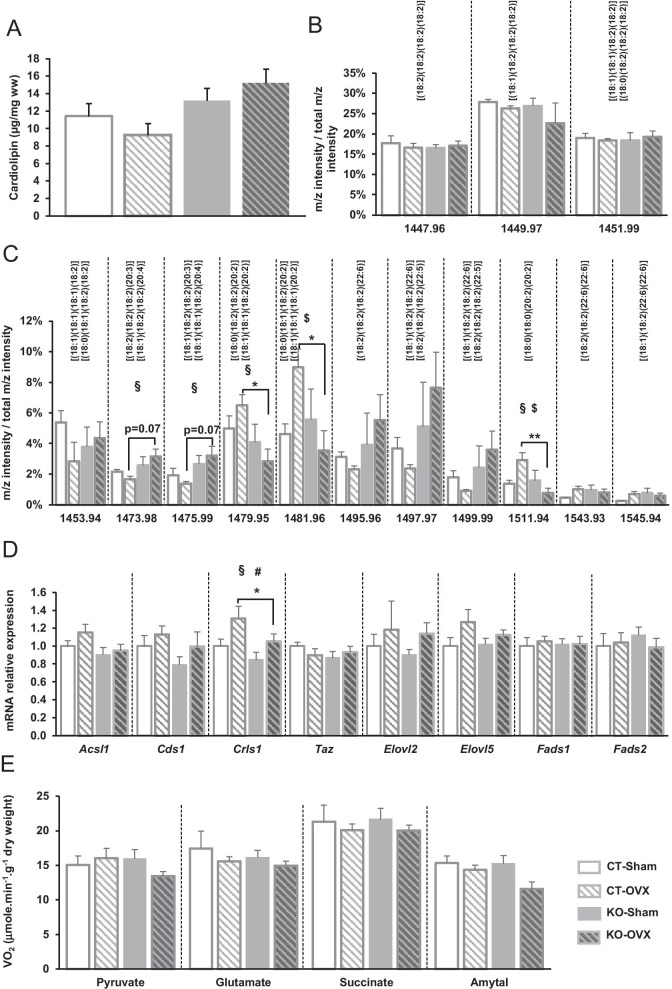
Cardiolipin profile and oxidative capacities 16 weeks after cardiac-specific *Ampkα2* deletion in control (Sham) and ovariectomized (OVX) female mice. **A** Total cardiolipin content. **B** Relative content of major cardiolipin species. **C** Relative content of minor cardiolipin species. **D** Expression of genes related to cardiolipin biosynthesis and maturation: acyl-CoA synthetase long-chain family member 1 (Acsl1), CDP-diacylglycerol synthase 1 (Cds1), cardiolipin synthase 1 (Crls1), tafazzin (Taz), elongation of very long chain fatty acids-like 2 (Elovl2), elongation of long chain fatty acids (Elovl5), fatty acid desaturase 1 (Fads1) and fatty acid desaturase 2 (Fads2). **E** Rate of respiration after successive addition of pyruvate (1 mM), glutamate (10 mM), succinate (15 mM), amytal (1 mM). (*n* = 4 to 7 per experimental group). ANOVA: #*p* ≤ 0.05 for the OVX effect; §*p* < 0.05 for the genotype effect, $*p* < 0.05 interaction. Post hoc Tukey test: *p < 0.05, **p < 0.01 CT-OVX vs KO-OVX

Since the loss of AMPKα2 led to drastic changes in CL profile in males, mitochondrial membrane CL content/profile has been assessed after ovary excision. Whereas the total CL content and the major CL species were not affected by the genotype or the hormone deficiency (Fig. 9A and B), many minor CL species were impacted by ovariectomy in a genotype-dependent manner. Indeed, even if no significant changes were noticed when each OVX group was compared to its respective sham group (Fig. 9C), the proportion of CL with eicosadienoic acid (20:2) and CL containing eicosatrienoic acid (20:3) or eicosatetraenoic acid (20:4) slightly changed following ovariectomy. Notably, the mild modulations noticed in KO-OVX mice compared to KO-sham group were systematically the opposite of the slight alterations displayed by CT-OVX group when compared to CT-sham one. This led to significant lower proportion of CL with eicosadienoic acid (20:2) and a trend towards a higher proportion of CL containing eicosatrienoic acid (20:3) or eicosatetraenoic acid (20:4) in KO-OVX mice than in CT-OVX ones (Fig. 9C), mimicking the pattern observed in KO males versus CT males (Fig. 6C). These OVX induced-CL profile modulations could thus be under the control of AMPK and this was confirmed by the two-way ANOVA statistical analysis which indicated an interaction between surgery and genotype for the variations of the proportion of CL with eicosadienoic acid (20:2) (Fig. 9C). These differences between OVX groups was hardly explained by the expression of the enzymes involved in the synthesis/maturation of the CL since only the expression of *Crls1* was significantly lower in KO-OVX group while the expression of the other enzymes of this biosynthesis pathway was neither affected by surgery nor by genotype (Fig. 9D). Although the alterations observed in mitochondrial membrane composition in KO-OVX mice is reminiscent of what was described in male *Ampkα2ciKO* mice, they were less marked than in males and seemed not to be sufficient to clearly affect mitochondrial function since mitochondrial respiratory assay did not show significant alterations in KO-OVX group in comparison with the other groups (Fig. 9E).

## Discussion

Although the role of AMPK has largely been studied for many years, its role in cardiac physiology and pathophysiology is still not completely understood. With the aim of clarifying the place of this kinase in cardiomyocyte homeostasis, we generated an original inducible cardiac-specific *Ampkα2* knockout mouse model using Cre recombinase. Of note, none of the deleterious effects on cardiac function observed in males were due to a potential toxicity of Cre recombinase since our team already demonstrated that the tamoxifen conditions used for inducing gene deletion do not impact cardiac function in αMHC-MerCreMer mice [37]. Using this inducible cardiac-specific *Ampkα2* knockout mouse model, we show that specific cardiac deficiency of AMPKα2 at adult age in males (1) did not induce cardiac hypertrophy; (2) led to a mild left ventricular dysfunction; (3) resulted in the development of cardiac fibrosis; (4) reduced complex I-driven respiration without changes in mitochondrial mass or in in vitro complex I activity; (5) was associated with a rearrangement of the CL species and a reduced integration of complex I into the ETC supercomplexes. Importantly, the effects of the cardiac-specific *Ampkα2* deletion observed in males were not found in females at basal state, suggesting that alternative pathways to AMPK are active in female mice. Ovariectomy of female KO mice suggest that their lower sensitivity to cardiac *Ampkα2* deletion could partly be explained by the blunting effects of female hormones on the development of cardiac fibrosis and by a role in the maturation of cardiolipins; however alterations in these females remained quite mild since they displayed normal mitochondrial function and were asymptomatic as regard to systolic function.

Owing to the AMPK structure based on the assembly of several subunits, suppression of AMPK activity in genetically modified animal models can result from different strategies. Many *Ampk* null mice have already been generated using KO or dominant negative technologies targeting one or the other subunit of AMPK and gave variable results on AMPK activity and compensatory mechanisms between the diverse isoforms [24, 38–40]. In the present murine model of cardiac specific and inducible *Ampkα2* deletion, the AMPKα2 protein content in LV was drastically decreased and was associated with a reduction in phosphorylated AMPK level. Yet, phosphorylation of ACC, a direct target of AMPK, was not modified. This result which can be surprising at first sight, can actually be explained by the fact that the experiments have been done in non-stressing conditions which are not expected to induce strong AMPK activity and consequently a high ACC phosphorylation level. Furthermore, this indicates that the tiny residual amount of AMPKα2 together with AMPKα1, the expression of which was not changed in the present model, could maintain the amount of phosphorylated ACC at basal level. Indeed, ACC is a common target of both AMPKα isoforms and a small amount of AMPKα1/2 could be sufficient to ensure phosphorylation status of this enzyme when the animal is not subjected to stress [41]. Nevertheless, as AMPK phosphorylates many other proteins and as the affinity of AMPKα1 and AMPKα2 for their targets differs and gives them a relative specificity [6], the drastic decrease in AMPKα2 content in *Ampkα2ciKO* males may impact the signaling pathways downstream of AMPK because the residual AMPKα2 could not ensure phosphorylation of all the targets, some of which would not be phosphorylated by AMPKα1 as efficiently as by AMPKα2.

In male *Ampkα2ciKO* mice, the modest alterations of several cardiac contractile parameters (EF, FS, LVIDd, LVIDs) suggest a mild left ventricular systolic dysfunction which is in accordance with the cardiac impairment described in global and constitutive *Ampkα2* deletion [17, 25, 39]. The degradation of cardiac function was clearly evidenced 7 weeks after deletion, got worst as the mice deleted for *Ampkα2* got older, but was not associated with important anatomical changes when compared to control. Several studies showed narrow links between AMPK and protein synthesis regulation [42, 43] and suggested an anti-hypertrophic action of AMPK under various stresses [39, 44, 45]. However, the present work did not show any modifications of the cardiac mass neither in *Ampkα2ciKO* male nor in *Ampkα2ciKO* female mice when the animals were not subjected to any stress. This result, which incidentally recalls what was observed in other dominant negative or global *Ampkα2* KO mice [17, 39], suggests that this AMPK isoform does not play critical role in cardiac hypertrophy/growth at basal state. For the understanding of this study, it seems important to note that the absence of cardiac dysfunction after 16 weeks of *Ampkα2* deletion in females probably does not mean that females are totally insensible to AMPKα2 loss. Indeed, although not statistically significant the slight increase in *Bnp* expression in *Ampkα2ciKO* females after 16 weeks of deletion could suggest that females were not fully protected from this deletion but rather more resistant. It cannot be excluded that these females would develop significant cardiac dysfunction at a later age and this point will require further investigations.

Interestingly, the ventricular dysfunction observed in males was associated with a significant increase of myocardial fibrosis that was correlated with EF. Although this could imply that fibrosis affects ventricular mechanical properties and function in this model, the fact that KO-OVX mice exhibited a comparable cardiac fibrosis level without any changes in cardiac function compels us to consider that fibrosis is not the only factor explaining cardiac dysfunction in males. Be that as it may, the development of fibrosis in *Ampkα2ciKO* male and in KO-OVX female mice could suggest that AMPKα2 is linked to fibrosis. It is generally thought that the cardiac fibrotic response is essentially mediated by cardiac fibroblasts; yet in this cardiac-specific *Ampkα2* KO model, *Ampkα2* was only deleted in cardiomyocytes and it could be suggested that this deletion would stimulate cardiac fibrotic response by cardiac fibroblasts through communication between both cell types. Even though the link between AMPK and cardiac fibrosis has already been suggested since the activation of AMPK can suppress fibrosis induced by various stresses (for review see [16]), regulation of the fibrotic response by AMPKα2 is still poorly understood, on the contrary to the role of AMPKα1, the major AMPK isoform in cardiac fibroblasts, which has clearly been demonstrated to control the proliferation of cardiac fibroblasts and the development of fibrosis especially through the TGF-β1/p38 axis [15]. From another side, it cannot be excluded that fibrosis observed in males was linked to mitochondrial dysfunction since it has recently been shown that oxidative stress plays a key role in myocardial fibrosis development [46, 47] and excessive ROS production is a well-known feature of altered mitochondria. Nevertheless, cardiac fibrosis in OVX group was not associated with clear alteration of electron transfer chain, rather suggesting that cardiac fibrosis was an earlier event than important mitochondrial perturbations in these mice and giving weight to the hypothesis of a link between AMPK and fibrosis. It could also reasonably be proposed that mitochondrial dysfunction could at least secondarily participates in fibrosis establishment in this model, all the more so mitochondrial dysfunction could be at the origin of cell death within the myocardium which is known to trigger reparative fibrosis to prevent rupture of the ventricular wall.

In this study, cardiac *Ampkα2* deletion provoked mitochondrial complex I-driven respiration dysfunction only in male LV. This was not associated with any significant changes in maximal mitochondrial oxidative capacity and mitochondrial mass, even though gene expression of several markers of mitochondrial biogenesis were significantly reduced (*Nrf-2* and *Cox1*) or tended to be lower (*ERRα*, *Tfam*, *Pparα* and *Cox4*) in KO. This is in accordance with the observation made in the mouse model of global and constitutive deletion of *Ampkα2* [17] and suggests that this kinase would only play a minor role in mitochondrial biogenesis regulation in the non-stressed heart. Similarly, cardio-specific or constitutive deletion of *Ampkα2* led to complex I-driven respiration impairment without any decrease in complex I enzymatic activity measured in total heart extract, thereby suggesting an alteration of in situ regulation of this complex.

It is known that the in situ activity of ETC complexes can be affected by the phospholipid content of inner mitochondrial membrane and that complex I is particularly sensitive to its microenvironment [48]. Cardiolipin, a phospholipid which represents 15 to 20% of total mitochondrial phospholipid is essentially found in the inner membrane of the mitochondria [17]. It is formed of two phosphate moieties connected with a glycerol backbone. It thus contains four acyl groups and carries negative charges. Cardiolipin mainly contain (18:2)4 acyl chains, but the length and composition of acyl chains can vary enormously. In the global constitutive *Ampkα2* KO mice, the alteration of complex I-driven respiration was associated with a clear reduction in LV content of CL [17]. Given that CLs play a key role in many processes of mitochondrial metabolism [49], this suggested that the decrease in CL could partly explain the ETC defects observed in this model. In the present work, cardiac-specific *Ampkα2* deletion in males induced a decrease in the expression of several key enzymes of CL biosynthesis (*Cds1, Acsl1* and *Crls1*) and substantial CL rearrangement even though the total CL content was similar to control. The difference between global constitutive *Ampkα2* KO mice and the present model could come from the different timing and duration of the deletion, from the beginning of in utero life in the constitutive KO *versus* a loss of AMPK starting in the adult life in *Ampkα2ciKO* mice, from an established pool of CL in mitochondria. Nevertheless, our present study and a previous one by our team on doxorubicin-induced cardiomyopathy further bring evidence that AMPK loss has an impact on mitochondrial CL profile [17, 21]. Recently, it has been shown that CL remodeling alters lipid membrane properties and assembly of complex subunits [50]. The increase in CL containing docosahexaenoic acyl chain (22:6) in KO males is particularly interesting as it has been reported that a higher proportion of tetradocosahexaenoyl-CL in the heart disrupts the formation of microdomains and phospholipid–protein contacts regulating the mitochondrial enzymes, especially complex I and IV [51], thereby demonstrating a strong link between CL and ETC. Rather than existing as individual complexes, respiratory chain complexes physically interact in highly organized structures allowing functional link between ETC components resulting in a more efficient electron transfer [52]. Given that CL create the environment required for the assembly of complex subunits and the formation of supercomplexes allowing an efficient complex-I-linked and complex II-linked mitochondrial respiration [53], the CL profile modulations observed in KO males is probably part of the mechanism leading to ETC alterations in our model.

The reduction in *Cds1* expression in *Ampkα2ciKO* mice suggests a role for AMPK signaling in the regulation of genes involved in CL biosynthesis. Interestingly, the expression of this enzyme has been shown to be under the control of PGC-1α/ERRα axis [54]. As a major regulator of PGC-1α, AMPK could thus modulate CL metabolism through this pathway. The trend towards a reduction in *Errα* expression in *Ampkα2ciKO* males in comparison with *Ampkα2*^*f/f*^ males is in favor of this hypothesis. The present study shows that this alteration of mitochondrial membrane CL composition was not observed in female KO mice in which complex I-driven respiration was similar to control. This once again highlights the connection between complex I activity and mitochondrial membrane CL composition [55, 56] and also suggests a sexual dimorphism in the regulation of mitochondrial membrane composition. Such a sexual dimorphism in CL composition has already been observed in rats [57]. Surprisingly, ablation of the ovaries in female *Ampkα2ciKO* mice did not affect the mitochondrial membrane CL composition as strongly as in males. Nevertheless, cardiac mitochondrial CL profile in ovariectomized females was modified according to a pattern reminding the rearrangements observed in *Ampkα2ciKO* males, strengthening the idea that female hormones normally participate in the maintenance of inner mitochondrial CL composition and compensate for the lack of *Ampkα2* in KO-sham females. Even so, the CL alterations in KO-OVX mice are quite mild and appear insufficient to impact mitochondrial function as in the males, suggesting that the duration of female hormone deficiency was not sufficient to totally suppress the higher resistance of the *Ampkα2ciKO* females. This could also be explained by the fact that OVX mice had surgery at the age of 7 weeks and a first impregnation with female hormones cannot be excluded as 6-week-old mice may be able to reproduce. These mice could have been exposed to female hormones during one or two weeks and it could interfere with the development of the phenotype of *Ampkα2ciKO* mice and explain the milder effect of *Ampkα2* deletion in OVX mice than in males. In addition, even after ovariectomy, females continue to display a different global morphology than males and are not exposed to high level of male hormones. The fact that *Ampkα2ciKO* OVX females did not perfectly reproduced the CL rearrangement observed in males may not be that surprising since male hormones could also differently regulate CL biosynthesis [58]. In the heart, the profound disparities in CL content/species between male and female rats subjected to doxorubicin treatment pleads in favor of a sexual dimorphism in CL metabolism [57]. Together these studies suggest that the role of male and female hormones in CL biosynthesis/maturation requires further investigations. So far, the role of AMPK in the sexual dimorphism of CL metabolism is still elusive. Although it might have been interesting to study the reversibility of the *Ampkα2* deletion-induced alterations in OVX females by 17-β estradiol treatment to better understand the underlying phenomena, the present study clearly confirms that AMPK is involved in CL biosynthesis and remodeling in a sex-dependent manner.

CLs play many roles in mitochondrial membrane organization and are crucial for mitochondrial supercomplex formation [59]. In male *Ampkα2* KO mice, the proportion of complex I not enclosed in supercomplexes was increased and these mice displayed less cardiac complex-I-containing supercomplexes. When considering the fact that neither mitochondrial respiration nor CLs profile nor the amount of complex-I-containing supercomplexes were significantly modulated in female KO mice, one can suggest that the concomitant alterations of ETC functioning, mitochondrial membrane CL composition and supercomplex disorganization in males, together with modest systolic dysfunction are in no way coincidental. In X-linked Barth syndrome induced by a mutation in the gene encoding tafazzin, an enzyme responsible for CL maturation, mature CLs are lost and this leads to an instability of respiratory chain supercomplexes which affects complex I activity [60], thereby highlighting the importance of CLs for complex I-containing supercomplexes formation and function. In male *Ampkα2* KO mice, the alterations of CL profile and complex I-containing supercomplexes formation undeniably affects the ETC functioning and this at least explains in part the alteration of complex-I-driven respiration observed in this model.

## Limitations of the study

The fact that *Ampkα2* deletion is induced by injections of tamoxifen which interacts with estrogen receptor could be a limitation of this study that was focused on sex differences. However, the tamoxifen dose used was chosen after a long period of development and study of the literature to determine the lowest dose of tamoxifen to achieve an optimal level of deletion of *Ampkα2* and limit side-effects. Thus, each mouse was injected with only two 40 mg/kg doses which correspond to 2 doses of 1 mg tamoxifen for a 25 g mouse. A recent study testing the clearance of tamoxifen in mice [61] showed that after high dose tamoxifen (5-days treatment with 3 mg or 6 mg tamoxifen daily (intraperitoneal)) the concentration of tamoxifen in serum is very low 10 days after the last injection. The doses used in this study were 7.5 or 15 times higher than in the present study, it can be assumed that our mice were not exposed to tamoxifen more than a few days. As most of the measurements realized in our study have been done 16 weeks after the last tamoxifen injection, the potential impacts of tamoxifen through estrogen signaling interaction on the produced results were probably limited.

Naturally, AMPK is involved in many signaling pathways and the phenotype of *Ampkα2ciKO* mice was the result of a set of perturbations. We did not extensively study AMPK roles which are particularly intricate and there were undoubtedly many other disturbances that had not been uncovered in this work. Nevertheless, this study has the merit of highlighting a poorly understood role for AMPK, which partly explains the disturbances in energy metabolism observed in this model and which certainly played its part in the phenotype resulting from the deletion of *Ampkα2* in the heart.

## Perspectives and significance

Cardiac-specific inducible *Ampkα2* deletion model proved helpful in deciphering the role of AMPK in basal conditions. In this model, *Ampkα2* deletion induced a progressive cardiac dysfunction associated with cardiac fibrosis and a mitochondrial dysfunction associated with cardiolipin remodeling in males only, thereby suggesting a role for AMPKα2 in these processes. Although the involvement of AMPKα2 in the regulation of myocardial fibrosis and cardiolipin biosynthesis and maturation requires further studies, our results highlight a higher dependence on AMPK signaling for these processes in males, either due to the absence of female hormones protection or to counteract the action of male hormones, and that may contribute to the known difference in cardiovascular risk and outcome between sexes.

## Data Availability

The data that support the findings of this study are available from the corresponding author upon reasonable request.

## Abbreviations

ACC: Acetyl-CoA carboxylase
AMPK: AMP-activated protein kinase
*Ampkα2ciKO*: *Ampkα2* Cardiac-specific inducible mice
CL: Cardiolipin
COX: Cytochrome c oxidase
CS: Citrate synthase
CT: Control mice
EF: Ejection fraction
ETC: Electron transfer chain
FS: Fractional shortening
HF: Heart failure
LV: Left ventricle
OVX: Ovariectomy
ROS: Reactive oxygen species

## References

[1] Hardie DG, Carling D (1997). The AMP-activated protein kinase–fuel gauge of the mammalian cell?. Eur J Biochem.

[2] Cheung PCF, Salt IP, Davies SP, Hardie DG, Carling D (2000). Characterization of AMP-activated protein kinase gamma-subunit isoforms and their role in AMP binding. Biochem J.

[3] Sambandam N, Lopaschuk GD (2003). AMP-activated protein kinase (AMPK) control of fatty acid and glucose metabolism in the ischemic heart. Prog Lipid Res.

[4] Dolinsky VW, Dyck JRB (2006). Role of AMP-activated protein kinase in healthy and diseased hearts. Am J Physiol-Heart C.

[5] Hawley SA, Davison M, Woods A, Davies SP, Beri RK, Carling D (1996). Characterization of the AMP-activated protein kinase kinase from rat liver and identification of threonine 172 as the major site at which it phosphorylates AMP-activated protein kinase. J Biol Chem.

[6] Woods A, Salt I, Scott J, Hardie DG, Carling D (1996). The alpha1 and alpha2 isoforms of the AMP-activated protein kinase have similar activities in rat liver but exhibit differences in substrate specificity in vitro. FEBS Lett.

[7] Hurley RL, Anderson KA, Franzone JM, Kemp BE, Means AR, Witters LA (2005). The Ca2+/calmodulin-dependent protein kinase kinases are AMP-activated protein kinase kinases. J Biol Chem.

[8] Hardie DG, Ashford ML (2014). AMPK: regulating energy balance at the cellular and whole body levels. Physiology (Bethesda).

[9] Russell RR, Bergeron R, Shulman GI, Young LH. Translocation of myocardial GLUT-4 and increased glucose uptake through activation of AMPK by AICAR. Am J Physiol-Heart C. 1999;277(2):H643–H9. <Go to ISI>://WOS:000081865800026. 10.1152/ajpheart.1999.277.2.H643 10444490

[10] Marsin AS, Bertrand L, Rider MH, Deprez J, Beauloye C, Vincent MF (2000). Phosphorylation and activation of heart PFK-2 by AMPK has a role in the stimulation of glycolysis during ischaemia. Curr Biol.

[11] Kudo N, Barr AJ, Barr RL, Desai S, Lopaschuk GD (1995). High-rates of fatty-acid oxidation during reperfusion of ischemic hearts are associated with a decrease in Malonyl-Coa levels due to an increase in 5'-Amp-activated protein-kinase inhibition of Acetyl-Coa carboxylase. J Biol Chem.

[12] Hue L, Beauloye C, Bertrand L, Horman S, Krause U, Marsin AS (2003). New targets of AMP-activated protein kinase. Biochem Soc T.

[13] Song P, Zou MH (2012). Regulation of NAD(P)H oxidases by AMPK in cardiovascular systems. Free Radic Biol Med.

[14] Balteau M, Van Steenbergen A, Timmermans AD, Dessy C, Behets-Wydemans G, Tajeddine N (2014). AMPK activation by glucagon-like peptide-1 prevents NADPH oxidase activation induced by hyperglycemia in adult cardiomyocytes. Am J Physiol Heart Circ Physiol.

[15] Noppe G, Dufeys C, Buchlin P, Marquet N, Castanares-Zapatero D, Balteau M (2014). Reduced scar maturation and contractility lead to exaggerated left ventricular dilation after myocardial infarction in mice lacking AMPKalpha1. J Mol Cell Cardiol.

[16] Daskalopoulos EP, Dufeys C, Bertrand L, Beauloye C, Horman S (2016). AMPK in cardiac fibrosis and repair: Actions beyond metabolic regulation. J Mol Cell Cardiol.

[17] Athea Y, Viollet B, Mateo P, Rousseau D, Novotova M, Garnier A (2007). AMP-activated protein kinase alpha2 deficiency affects cardiac cardiolipin homeostasis and mitochondrial function. Diabetes.

[18] He Q, Wang M, Harris N, Han XL (2013). Tafazzin knockdown interrupts cell cycle progression in cultured neonatal ventricular fibroblasts. Am J Physiol-Heart C.

[19] He QA (2010). Tafazzin knockdown causes hypertrophy of neonatal ventricular myocytes. Am J Physiol-Heart C.

[20] Jussupow A, di Luca A, Kaila VRI (2019). How cardiolipin modulates the dynamics of respiratory complex I. Sci Adv.

[21] Moulin M, Piquereau J, Mateo P, Fortin D, Rucker-Martin C, Gressette M (2015). Sexual dimorphism of doxorubicin-mediated cardiotoxicity: potential role of energy metabolism remodeling. Circ Heart Fail.

[22] Regitz-Zagrosek V, Kararigas G (2017). Mechanistic pathways of sex differences in cardiovascular disease. Physiol Rev.

[23] Russell RR, Li J, Coven DL, Pypaert M, Zechner C, Palmeri M (2004). AMP-activated protein kinase mediates ischemic glucose uptake and prevents postischemic cardiac dysfunction, apoptosis, and injury. J Clin Investig.

[24] Zarrinpashneh E, Carjaval K, Beauloye C, Ginion A, Mateo P, Pouleur AC (2006). Role of the alpha2-isoform of AMP-activated protein kinase in the metabolic response of the heart to no-flow ischemia. Am J Physiol Heart Circ Physiol.

[25] Carvajal K, Zarrinpashneh E, Szarszoi O, Joubert F, Athea Y, Mateo P (2007). Dual cardiac contractile effects of the alpha2-AMPK deletion in low-flow ischemia and reperfusion. Am J Physiol Heart Circ Physiol.

[26] Sasaki H, Asanuma H, Fujita M, Takahama H, Wakeno M, Ito S (2009). Metformin prevents progression of heart failure in dogs: role of AMP-activated protein kinase. Circulation.

[27] Siasos G, Tsigkou V, Kosmopoulos M, Theodosiadis D, Simantiris S, Tagkou NM (2018). Mitochondria and cardiovascular diseases-from pathophysiology to treatment. Ann Transl Med.

[28] Viollet B, Andreelli F, Jorgensen SB, Perrin C, Geloen A, Flamez D (2003). The AMP-activated protein kinase alpha2 catalytic subunit controls whole-body insulin sensitivity. J Clin Investig.

[29] Kuznetsov AV, Veksler V, Gellerich FN, Saks V, Margreiter R, Kunz WS (2008). Analysis of mitochondrial function in situ in permeabilized muscle fibers, tissues and cells. Nat Protoc.

[30] Wharton DC, Tzagoloff A (1967). Cytochrome oxidase from beef heart mitochondria. Methods Enzymol.

[31] Veksler VI, Kuznetsov AV, Anflous K, Mateo P, van Deursen J, Wieringa B (1995). Muscle creatine kinase-deficient mice II Cardiac and skeletal muscles exhibit tissue-specific adaptation of the mitochondrial function. J Biol Chem.

[32] Wittig I, Braun HP, Schagger H (2006). Blue native PAGE. Nat Protoc.

[33] Rimbaud S, Ruiz M, Piquereau J, Mateo P, Fortin D, Veksler V (2011). Resveratrol improves survival, hemodynamics and energetics in a rat model of hypertension leading to heart failure. PLoS ONE.

[34] Rimbaud S, Sanchez H, Garnier A, Fortin D, Bigard X, Veksler V (2009). Stimulus specific changes of energy metabolism in hypertrophied heart. J Mol Cell Cardiol.

[35] Folch J, Lees M, Sloane Stanley GH (1957). A simple method for the isolation and purification of total lipides from animal tissues. J Biol Chem.

[36] Imbert L, Ramos RG, Libong D, Abreu S, Loiseau PM, Chaminade P (2012). Identification of phospholipid species affected by miltefosine action in *Leishmania donovani* cultures using LC-ELSD, LC-ESI/MS, and multivariate data analysis. Anal Bioanal Chem.

[37] Sanz MN, Grimbert L, Moulin M, Gressette M, Rucker-Martin C, Lemaire C (2019). Inducible cardiac-specific deletion of Sirt1 in male mice reveals progressive cardiac dysfunction and sensitization of the heart to pressure overload. Int J Mol Sci.

[38] Viollet B, Foretz M (2016). Animal models to study AMPK. EXS.

[39] Zhang P, Hu X, Xu X, Fassett J, Zhu G, Viollet B (2008). AMP activated protein kinase-alpha2 deficiency exacerbates pressure-overload-induced left ventricular hypertrophy and dysfunction in mice. Hypertension.

[40] Sung MM, Zordoky BN, Bujak AL, Lally JS, Fung D, Young ME (2015). AMPK deficiency in cardiac muscle results in dilated cardiomyopathy in the absence of changes in energy metabolism. Cardiovasc Res.

[41] Chen S, Zhu P, Guo HM, Solis RS, Wang Y, Ma Y (2014). Alpha1 catalytic subunit of AMPK modulates contractile function of cardiomyocytes through phosphorylation of troponin I. Life Sci.

[42] Inoki K, Zhu T, Guan KL (2003). TSC2 mediates cellular energy response to control cell growth and survival. Cell.

[43] Gwinn DM, Shackelford DB, Egan DF, Mihaylova MM, Mery A, Vasquez DS (2008). AMPK phosphorylation of raptor mediates a metabolic checkpoint. Mol Cell.

[44] Tian R, Musi N, D'Agostino J, Hirshman MF, Goodyear LJ (2001). Increased adenosine monophosphate-activated protein kinase activity in rat hearts with pressure-overload hypertrophy. Circulation.

[45] Turdi S, Kandadi MR, Zhao J, Huff AF, Du M, Ren J (2011). Deficiency in AMP-activated protein kinase exaggerates high fat diet-induced cardiac hypertrophy and contractile dysfunction. J Mol Cell Cardiol.

[46] Rajgarhia A, Ayasolla KR, Zaghloul N, Da Lopez JM, Miller EJ, Ahmed M (2021). Extracellular superoxide dismutase (EC-SOD) Regulates gene methylation and cardiac fibrosis during chronic hypoxic stress. Front Cardiovasc Med..

[47] Li J, Ding H, Li Y, Zhou H, Wang W, Mei Y (2021). Alarin alleviated cardiac fibrosis via attenuating oxidative stress in heart failure rats. Amino Acids.

[48] Schlame M, Rua D, Greenberg ML (2000). The biosynthesis and functional role of cardiolipin. Prog Lipid Res.

[49] Paradies G, Paradies V, Ruggiero FM, Petrosillo G (2019). Role of cardiolipin in mitochondrial function and dynamics in health and disease: molecular and pharmacological aspects. Cells.

[50] El-Hafidi M, Correa F, Zazueta C (2020). Mitochondrial dysfunction in metabolic and cardiovascular diseases associated with cardiolipin remodeling. Biochim Biophys Acta Mol Basis Dis.

[51] Sullivan EM, Pennington ER, Sparagna GC, Torres MJ, Neufer PD, Harris M (2018). Docosahexaenoic acid lowers cardiac mitochondrial enzyme activity by replacing linoleic acid in the phospholipidome. J Biol Chem.

[52] Letts JA, Sazanov LA (2017). Clarifying the supercomplex: the higher-order organization of the mitochondrial electron transport chain. Nat Struct Mol Biol.

[53] Maekawa S, Takada S, Nambu H, Furihata T, Kakutani N, Setoyama D (2019). Linoleic acid improves assembly of the CII subunit and CIII2/CIV complex of the mitochondrial oxidative phosphorylation system in heart failure. Cell Commun Signal.

[54] Lai L, Wang M, Martin OJ, Leone TC, Vega RB, Han X (2014). A role for peroxisome proliferator-activated receptor gamma coactivator 1 (PGC-1) in the regulation of cardiac mitochondrial phospholipid biosynthesis. J Biol Chem.

[55] Drose S, Zwicker K, Brandt U (2002). Full recovery of the NADH:ubiquinone activity of complex I (NADH:ubiquinone oxidoreductase) from Yarrowia lipolytica by the addition of phospholipids. Biochem Biophys Acta.

[56] Paradies G, Petrosillo G, Pistolese M, Ruggiero FM (2002). Reactive oxygen species affect mitochondrial electron transport complex I activity through oxidative cardiolipin damage. Gene.

[57] Moulin M, Solgadi A, Veksler V, Garnier A, Ventura-Clapier R, Chaminade P (2015). Sex-specific cardiac cardiolipin remodelling after doxorubicin treatment. Biol Sex Differ.

[58] Acaz-Fonseca E, Ortiz-Rodriguez A, Lopez-Rodriguez AB, Garcia-Segura LM, Astiz M (2017). Developmental sex differences in the metabolism of cardiolipin in mouse cerebral cortex mitochondria. Sci Rep.

[59] Mileykovskaya E, Dowhan W (2014). Cardiolipin-dependent formation of mitochondrial respiratory supercomplexes. Chem Phys Lipids.

[60] McKenzie M, Lazarou M, Thorburn DR, Ryan MT (2006). Mitochondrial respiratory chain supercomplexes are destabilized in Barth Syndrome patients. J Mol Biol.

[61] Donocoff RS, Teteloshvili N, Chung H, Shoulson R, Creusot RJ (2020). Optimization of tamoxifen-induced Cre activity and its effect on immune cell populations. Sci Rep.

